# The Balance of TNF Mediated Pathways Regulates Inflammatory Cell Death Signaling in Healthy and Diseased Tissues

**DOI:** 10.3389/fcell.2020.00365

**Published:** 2020-05-21

**Authors:** Joshua D. Webster, Domagoj Vucic

**Affiliations:** Departments of Pathology and Early Discovery Biochemistry, Genentech, South San Francisco, CA, United States

**Keywords:** TNF, RIP1 (RIPK1), RIP3 kinase, NEMO, necroptois, apoptosis, RIPK1 inhibitors

## Abstract

Tumor necrosis factor alpha (TNF; TNFα) is a critical regulator of immune responses in healthy organisms and in disease. TNF is involved in the development and proper functioning of the immune system by mediating cell survival and cell death inducing signaling. TNF stimulated signaling pathways are tightly regulated by a series of phosphorylation and ubiquitination events, which enable timely association of TNF receptors-associated intracellular signaling complexes. Disruption of these signaling events can disturb the balance and the composition of signaling complexes, potentially resulting in severe inflammatory diseases.

## Structure of TNF and TNF Receptors

TNF is a type II transmembrane protein that is expressed at the plasma membrane as a trimer (Vassalli, 1992). Cleavage by tumor necrosis factor converting enzyme (TACE) can generate a soluble ligand that propagates signaling by binding to two receptors – TNFR1 (CD120a) and TNFR2 (CD120b) (Black et al., 1997; Moss et al., 1997). TNFR1 associates strongly with both membrane-bound and soluble TNF, while TNFR2 has much higher binding affinity for membrane-bound TNF (Grell et al., 1995). The extracellular region of both receptors has four homologous cysteine-rich domains (CRDs) but their intracellular regions are structurally different. The intracellular portion of TNFR1 possesses a protein-binding region called a death domain (DD), which allows homo- and hetero-typic interactions with other DD-containing proteins. TNFR2, on the other hand, has a TNF Receptor Associated Factor (TRAF) binding site that interacts with the TRAF family of signaling adaptors (Grell et al., 1995; Reddy et al., 2000). The distinct expression profiles and stark difference in the intracellular regions of the TNF receptors greatly influence their physiological roles and cellular activity. Through engaging DD adaptors, broadly expressed TNFR1 can activate proliferative nuclear factor-kappa B (NF-κB) and mitogen-activated protein kinase (MAPK) signaling as well as cell death (Wallach et al., 1999; Sessler et al., 2013). On the other hand, TNFR2 is mostly expressed in immune and endothelial tissues. In addition, since it lacks a DD, TNFR2 cannot stimulate cell death, but uses TRAF recruitment to trigger NF-κB and MAPK activation (Wallach et al., 1999; Sessler et al., 2013). Due to its wide spectrum of cellular activities and ubiquitous expression, TNFR1 plays a prevailing role in TNF signaling and will be more extensively covered in this article.

## Activation of NF-κB and Mapk Signaling by TNF

**FIGURE 1:**
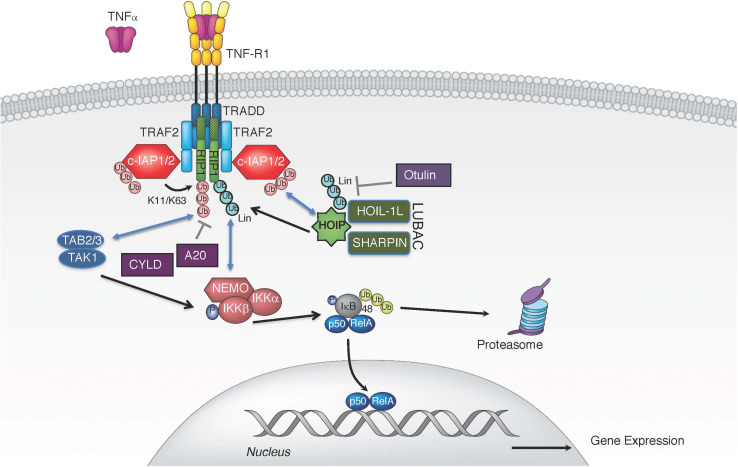
TNF induced canonical NF-κB pathway. TNF stimulation triggers the recruitment of TRADD, TRAF2, RIP1, and c-IAP1/2 to TNFR1. E3 ligases c-IAP1/2 polyubiquitinate themselves and RIP1 with K11 and K63 ubiquitin linkages, creating a platform for further recruitment of LUBAC. LUBAC mediates linear polyubiquitin, resulting in gene expression via the IKK complex. Several DUBs have been implicated in the regulation of TNFR1-associated complex I by removing linear (CYLD and OTULIN) or K63-linked polyubiquitin chains (A20 and CYLD).

Binding of TNF to TNFR1 triggers receptor trimerization and leads to the assembly of the TNFR1-associated signaling complex (complex I) (Figure 1). Within complex I, the adaptor proteins receptor interacting protein 1 (RIP1; RIPK1) and TNF receptor associated death domain (TRADD) are recruited to TNFR1 through their respective death domains (Micheau and Tschopp, 2003). TRADD then recruits adaptor proteins TRAF2 and TRAF5, which enables the engagement of the E3 ligases cellular inhibitors of apoptosis 1 and 2 (c-IAP1, c-IAP2) and subsequent ubiquitination of various components of complex I (Bertrand et al., 2008; Mahoney et al., 2008; Varfolomeev et al., 2008; Dynek et al., 2010). c-IAP1/2 promote self-ubiquitination and ubiquitination of RIP1 with K63-, K48-, and K11-linked chains, which are critical for TNFR1 complex I signaling (Bertrand et al., 2008; Mahoney et al., 2008; Varfolomeev et al., 2008; Dynek et al., 2010). K63-linked polyubiquitin chains conjugated onto c-IAP1/2 allow the recruitment of the linear ubiquitin chain assembly complex (LUBAC), which generates linear ubiquitin chains on several molecules including RIP1, TNFR1, LUBAC itself, and NF-κB essential modulator (NEMO) (Haas et al., 2009; Tokunaga et al., 2009; Ikeda et al., 2011; Tokunaga et al., 2011; Varfolomeev and Vucic, 2016). The LUBAC complex consists of adaptor proteins SHANK-associated RH-domain interactor (SHARPIN) and heme-oxidized IRP2 ubiquitin ligase 1 (HOIL-1L), and the E3 enzyme HOIL-1L-interacting protein (HOIP) (Tokunaga and Iwai, 2012). LUBAC produces linear or M1-linked ubiquitin chains by catalyzing a head-to-tail ubiquitination where a peptide bond between the N-terminal methionine of ubiquitin and the C-terminal glycine of the next ubiquitin is generated (Kirisako et al., 2006; Tokunaga et al., 2009). The diverse ensemble of polyubiquitin chains assembled during TNF-induced activation of NF-κB and MAPK includes, but is not limited to, K11, K48, K63, and linear chains (Dynek et al., 2010; Gerlach et al., 2011). This set of polyubiquitin chains provides a docking platform for the recruitment and retention of the signaling kinase complexes consisting of kinases IKKα and IKKβ (inhibitor of kappa B kinase 1 and 2) and the adaptor NEMO (IKKγ; IKK complex), and transforming growth factor beta-activated kinase 1 (TAK1) along with its partners, the K63-linked ubiquitin binding proteins TAK1-binding proteins 2 and 3 (TAB2/3) (Figure 1; Shim et al., 2005; Haas et al., 2009). The recruitment of kinase complexes leads to the activation of NF-κB and MAPK signaling and subsequent gene activation and expression of pro-inflammatory cytokines, such as interleukin 6 and 8 (IL-6, IL-8), and pro-survival proteins like c-IAP2 and the caspase-8 inhibitor cellular FLICE inhibitory protein (cFLIP) (Scheidereit, 2006).

The specific polyubiquitination pattern on RIP1 that keeps it in complex I for proper downstream activation of NF-κB and MAPKs is fine-tuned by the activation of E3 ligases, such as c-IAP1/2 (Bertrand et al., 2008; Mahoney et al., 2008; Varfolomeev et al., 2008; Silke and Vucic, 2014). The combined deletion of c-IAP1 and c-IAP2 in mice results in embryonic lethality and severe liver and intestinal damage in the adulthood, which can be rescued by TNFR1 knock-out or by TNF blockade, further emphasizing the functional and genetic relationship between these E3 ligases and TNF signaling (Moulin et al., 2012; Zhang J. et al., 2019). However, these complexes are also governed through negative regulation by deubiquitinases (DUBs). TNF stimulation also leads to transcriptional upregulation of deubiquitinases tumor necrosis factor alpha-induced protein 3 (TNFAIP3 or A20) and OTU domain DUB 7B (also known as Cezanne) whose DUB activity can dampen NF-κB signaling (Wertz et al., 2004; Enesa et al., 2008). A20 is an ubiquitin chain-binding enzyme that removes K63-linked ubiquitin chains from RIP1 to reduce NF-κB activation. Binding of linear ubiquitin chains via its zinc finger 7 motif is critical for A20’s recruitment to TNFR1 complex and suppression of inflammatory signaling (Tokunaga et al., 2012; Martens A. et al., 2020; Razani et al., 2020). Consequently, deletion of *A20* results in enhanced RIP1 ubiquitination and inflammation (Wertz et al., 2004; Zhou et al., 2016a). Cylindromatosis (CYLD) is another DUB whose recruitment to complex I can dampen NF-κB activation by hydrolyzing the K63-linked and linear polyubiquitin chains from the complex I components (Brummelkamp et al., 2003; Kovalenko et al., 2003; Trompouki et al., 2003). CYLD is recruited to the TNFR1 complex via the adaptor protein SPATA2, which binds the PUB (peptide:N-glycanase/UBA/X-containing protein) domain of HOIP through its PIM (PUB-interaction motif) (Elliott et al., 2016; Kupka et al., 2016; Schlicher et al., 2016; Wagner et al., 2016; Wei et al., 2017). Consequently, the absence of SPATA2, just like CYLD loss, enhances TNF stimulated NF-κB activation and dampens cell death (Elliott et al., 2016; Kupka et al., 2016; Schlicher et al., 2016; Wagner et al., 2016; Wei et al., 2017). Unlike these DUBs, which do not have a strict ubiquitin chain specificity, OTULIN (OTU deubiquitinase with linear specificity, also known as FAM105B or Gumby) binds the PUB domain of HOIP and selectively removes linear ubiquitin chains on LUBAC components thereby keeping uncontrolled TNF-associated inflammation in check (Fiil et al., 2013; Keusekotten et al., 2013; Elliott et al., 2014; Damgaard et al., 2016; Zhou et al., 2016b). Thus, a tightly controlled balance of E3 ligases and DUBs in the assembly and disassembly of diverse polyubiquitin chains on RIP1 and other signaling components is clearly needed for the appropriate level of signaling by complex I and corresponding gene activation.

### Cell Death Induction by TNF

**FIGURE 2:**
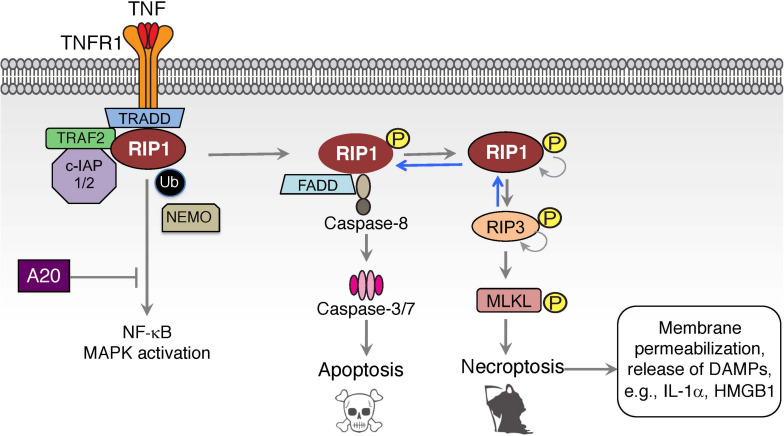
TNF induced cell death signaling. Inhibition of NF-κB and MAPK signaling can divert TNF-mediated signaling to the formation of an intracellular complex II centered on FADD and caspase-8 in a RIP1-dependent apoptotic cell death. This cell death pathway can be augmented by A20 or by the absence of the E3 ligases c-IAP1/2 or LUBAC, thereby eliminating the ubiquitination of complex I components and promoting the switch to complex II. Activation of RIP1-dependent cell death under caspase-8 inhibited or deficient conditions can lead to a necroptotic form of cell death that is mediated by kinase activity of RIP1 and RIP3, and results in the activation of MLKL and membrane permeabilization.

Dynamic changes of post-translational modifications of RIP1 and other components of TNFR1-associated signaling complexes can trigger a switch from inflammatory gene signaling to cell death via apoptosis or necroptosis (Figure 2). RIP1-dependent and RIP1-independent apoptotic signaling complexes can form in response to inhibited or altered NF-κB signaling (e.g., IKKβ or TAK1 inhibitors, genetic deletion of NF-κB) or the presence of transcriptional or translational inhibitors like actinomycin D or cycloheximide, respectively (Rubin et al., 1988; Micheau and Tschopp, 2003; Shan et al., 2018). A cytosolic complex II centered on TRADD recruits Fas-associated death domain (FADD) to activate caspase-8 and cause apoptotic cell death (Van Antwerp et al., 1996; Micheau and Tschopp, 2003; Wang et al., 2008). For RIP1-dependent apoptotic complex II, distinct ubiquitin modifications play a critical regulatory role in dictating the fate of cells. When E3 ligases c-IAP1/2 and LUBAC are degraded or absent, unmodified RIP1 dissociates from receptor-associated signaling complex I and associates with FADD through binding of their DDs (Micheau and Tschopp, 2003; Bertrand et al., 2008; Wang et al., 2008). FADD recruits pro-caspase 8 and/or its catalytically inactive homolog FLIP to form the death platform complex II using death effector domain (DED) interactions (Majkut et al., 2014). RIP1 dependent apoptosis can be further modulated by additional signaling proteins and E3 ligases (NEK1, APC11, LRKK2, and Cbl) that regulate the transition of RIP1 from complex I to complex II (Amin et al., 2018). Thus, the ubiquitination status of RIP1 determines the switch of RIP1 between pro-survival gene activation and cell death.

If caspase-8 is insufficiently activated or inhibited in complex II, RIP1 can autophosphorylate at S166 and bind RIP3 using their RIP homology interaction motifs (RHIM) leading to the formation of the necrosome (Sun et al., 2002; He et al., 2009; Wu et al., 2014; Laurien et al., 2020). Unlike complex I, where RIP1 kinase activity is dispensable, TNF stimulated necrosome formation is dependent on RIP1 kinase activity (Cho et al., 2009; He et al., 2009; Laurien et al., 2020). Within the necrosome, RIP3 undergoes auto-phosphorylation at S227 in human, and T231 and S232 in mouse RIP3 that is crucial for the execution of necroptotic cell death (Cho et al., 2009; He et al., 2009; Chen et al., 2013). Accordingly, genetic inactivation or chemical inhibition of their kinase functions blocks RIP1/3 dependent necroptotic cell death (Degterev et al., 2008; He et al., 2009; Newton et al., 2014). RIP3 phosphorylates necroptosis mediator mixed lineage kinase domain-like (MLKL) at residues T357 and S358 in human, and S345, S347, and T349 in mouse MLKL within its carboxy-terminal pseudokinase domain to execute necroptosis (Sun et al., 2012; Chen et al., 2013; Murphy et al., 2013; Khan et al., 2014; Wang et al., 2014; Rodriguez et al., 2016). How MLKL facilitates cell death is not entirely clear, but it does involve the disruption of cell membrane integrity. RIP3-phosphorylated MLKL undergoes a conformational change that exposes the N-terminal domain of MLKL, promoting its oligomerization and translocation to the membranes (Murphy et al., 2013; Dondelinger et al., 2014; Wang et al., 2014). Membrane associated MLKL may interfere directly with cell integrity by oligomeric insertion into the membrane thus causing membrane disruption/permeabilization/perturbation (Cai et al., 2014; Dondelinger et al., 2014; Hildebrand et al., 2014; Su et al., 2014; Wang et al., 2014). The pore-forming capacity of necroptosis results in a strong pro-inflammatory signal, a feature that places this cell death pathway at the core of many inflammatory and tissue-damage related diseases. However, RIP1 autophosphorylation can result in RIP1-dependent apoptosis as well, and a number of *in vivo* inflammatory animal models involve a mixture of RIP1-dependent apoptosis and necroptosis as we will describe later in this article (Patel et al., 2020; Webster et al., 2020).

## Disruption in TNF Signaling Ubiquitination Machinery in Patients With Immunodeficiency and Autoinflammation

**FIGURE 3:**
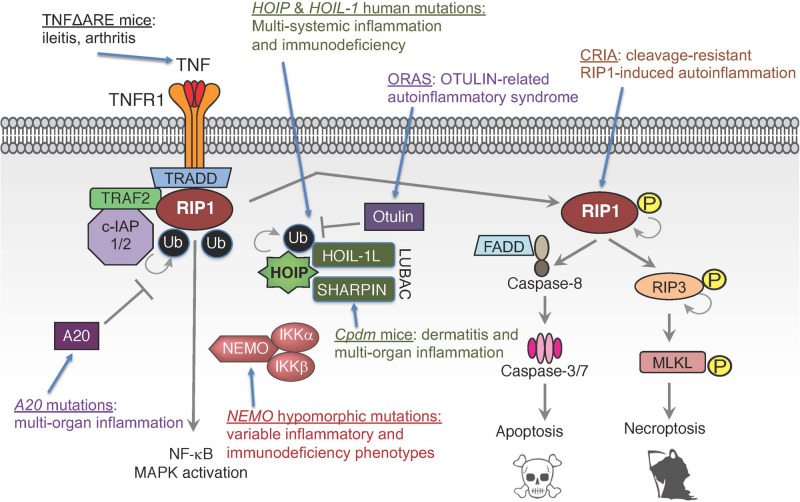
Human mutations and genetic models in TNF signaling pathways. Mutations in multiple components of TNF signaling leading to functional dysregulation of E3 ligase complex LUBAC, deubiquitinases A20 and OTULIN, ubiquitin-binding protein NEMO, ligand TNF or pro-death kinase RIP1 can cause inflammatory diseases and/or immunodeficiency.

While TNF’s importance in driving inflammatory diseases is well-established, the recent identification of patients with defects in TNF signaling components have shown the importance of tightly regulating this pathway and the potential consequences of its dysregulation (Manthiram et al., 2017; Oda and Kastner, 2017; Figure 3). *In vitro* and *in vivo* studies have suggested the critical role ubiquitin plays in TNF signaling, both in enabling signal complex formation and in protein degradation. For example, chronic proliferative dermatitis (*cpdm*) mice were originally characterized as a strain of C57BL/KaLawRij mice that developed eosinophilic dermatitis with epidermal hyperplasia, multi-systemic inflammation, and defects in lymphoid development (HogenEsch et al., 1993, 1999; Gijbels et al., 1996). Subsequent studies demonstrated that this phenotype was due to a spontaneous mutation in the *Sharpin* gene that resulted in diminished expression of SHARPIN and the other LUBAC components HOIP and HOIL-1L (Seymour et al., 2007; Gerlach et al., 2011; Tokunaga et al., 2011). Studies in these mice suggested that LUBAC mediated linear ubiquitination plays an important role in modulating inflammatory and cell death signaling downstream of TNFR1. However, the clinical validation of these observations has been more recently evident through the identification of patients with mutations in the genes encoding LUBAC components HOIP and HOIL-1L, and in mutations in the genes encoding the deubiquitinases OTULIN and A20.

Mutations in *HOIL-1L* were originally reported in 2012, in two families with immunodeficiency, as characterized by recurrent pyogenic infections, multi-systemic inflammation, and amylopectinosis (Boisson et al., 2012). The single described patient in the first family had a homozygous deletion of 2 nucleotides resulting in a premature stop codon. Patients in a second family had partial deletions of one allele and a nonsense point mutation in the second allele; suggesting an autosomal recessive mode of inheritance in both families. These *HOIL-1L* mutations caused an approximately 50% decrease in SHARPIN expression and near complete loss of HOIP expression. Loss of LUBAC expression resulted in impaired NF-κB activity in response to interleukin-1 beta (IL-1β) and, to a lesser extent, TNF in the patients’ fibroblasts. Similarly, the patients’ B cell responses to Toll-like receptor (TLR) 7 and 8 agonists, IL-1β, and CD40-ligand (CD40L) were also impaired. Interestingly, LUBAC deficiency had an opposing effect in monocytes. Specifically, monocytes had approximately four-fold increased IL-6 production following IL-1β stimulation and enhanced responses to TLR1 and 2 agonists (Boisson et al., 2012). While the patients described above presented with immune dysregulation, other *HOIL-1L* mutant patients present with primary myopathy and less frequent to no evidence of immune dysfunction (Nilsson et al., 2013; Wang et al., 2013). These patients presented with muscle weakness in adolescence that progresses over time, and a subset of patients develop dilated cardiomyopathy (Nilsson et al., 2013; Wang et al., 2013). Histologically, myofibers contain periodic acid-Schiff positive, amylase-resistant inclusions characteristic of polyglucosan (Nilsson et al., 2013). The reason that some patients with *HOIL-1L* mutations present for myopathy while others present for immunodeficiency is not fully understood, but the phenotype might be influenced by the location of the mutation in the gene (Nilsson et al., 2013).

An autosomal recessive missense mutation in *HOIP* has also been identified in one patient. This mutation resulted in a loss of HOIP protein and reduced levels of SHARPIN and HOIL-1L, resulting in LUBAC deficiency (Boisson et al., 2015). A second patient was identified with compound heterozygous *HOIP* polymorphisms. These polymorphisms caused alternative RNA splicing that resulted in truncated HOIP protein and LUBAC destabilization (Oda et al., 2019). Clinical and biochemical phenotypes in *HOIP* mutant patients mirrored patients with *HOIL-1L* mutations. Specifically, patients with mutations or polymorphisms in *HOIP* presented with multi-systemic inflammation and immunodeficiency characterized by recurrent infections, chronic diarrhea, and antibody deficiency (Boisson et al., 2015; Oda et al., 2019). Patients’ fibroblasts had blunted NF-κB responses to IL-1β and TNF, and their B cells had reduced response to CD40L (Boisson et al., 2015). Additionally, similar to HOIL-1L deficient patients, monocytes derived from these patients had increased response to IL-1β, resulting in elevated IL-6 and IL-1β production (Boisson et al., 2015).

Characterization of these patients confirms the critical role of LUBAC-mediated linear ubiquitination in NF-κB driven immune responses of fibroblasts and lymphocytes, and demonstrates that loss of this signaling has significant consequences including immunodeficiency and subsequent recurrent infections. However, the paradoxical increase in proinflammatory signaling in monocytes, which likely accounts for the concurrent multi-systemic inflammation, suggests that the role of LUBAC is dependent on cellular context and tight regulation of this pathway is critical to modulate inflammatory responses.

Deubiquitinases are critical to counter-regulate ubiquitin ligase activities. Just as c-IAP1/2 and LUBAC play fundamental roles in establishing signaling complexes downstream of the TNF receptor, deubiquitinases like OTULIN, CYLD, and A20 play equally important roles in modulating these complexes. Patients with reduced OTULIN expression due to autosomal recessive mutations develop fevers, dermatitis, and panniculitis (Damgaard et al., 2016, 2019; Zhou et al., 2016b; Nabavi et al., 2019). Comparable phenotypes are observed in patients with autosomal dominant mutations in the gene encoding A20, *TNFAIP3*. Specifically, these patients present with early onset systemic inflammation including arthritis, ophthalmitis, and oral and genital ulcers (Zhou et al., 2016a). Initial characterization of cells from affected patients revealed that TNF stimulated peripheral blood mononuclear cells (PBMCs) and fibroblasts from *OTULIN* and *TNFAIP3* mutant patients have increased NF-κB activity compared to controls and increased p38 phosphorylation in fibroblasts, which is associated with increased ubiquitination (Zhou et al., 2016a,b). These changes were correlated with increased serum cytokines in *TNFAIP3* mutant patients (Zhou et al., 2016a), and increased LPS-induced production of interferon-gamma, IL-1β, IL-6, IL-12, and IL-18 in whole blood samples of OTULIN deficient patients (Zhou et al., 2016b). Fibroblasts from a subsequently identified patient with a unique homozygous *OTULIN* mutation had reduced NFκB and p38 activity in response to TNF (Damgaard et al., 2019). The differences between these patients and their responses to TNF is unclear, since mutations characterized in both studies reportedly resulted in decreased OTULIN activity (Zhou et al., 2016b; Damgaard et al., 2019; Nabavi et al., 2019). Interestingly, although the later study found that OTULIN deficient fibroblasts were hypo-responsive to TNF and *shOTULIN* THP-1 cells were hyper-responsive to TNF, both cell types had increased susceptibility to cell death induced by the combination of TNF and cyclohexamide (Damgaard et al., 2019), suggesting that cell death is a common end product of dysregulation of this pathway. The clinical data and cellular characterization of HOIP, HOIL-1L, OTULIN, and A20 deficient patients highlight the essential role of ubiquitination in modulating TNF signaling. On the surface, the data suggest that too little ubiquitination (e.g., HOIP or HOIL-1L deficiency) results in dampening of the immune response, and persistent ubiquitination (e.g., OTULIN or A20 deficiency) causes autoinflammation. However, there are added complexities to this perspective, as noted in the increased IL-1β response in HOIP and HOIL-1L deficient monocytes.

Aside from autoinflammation, *A20* mutations also occur in approximately 12% of B cell lymphomas, with the highest incidence in mucosa-associated lymphoid tissue (MALT) lymphoma (Kato et al., 2009). Reconstitution of an A20 deficient lymphoma cell line with wild-type A20 resulted in decreased proliferation, increased apoptosis, and decreased NF-κB signaling. Similarly, A20 expressing cells transplanted into immunodeficient mice failed to develop tumors, as opposed to mock transfected cells, which developed tumors (Kato et al., 2009). Therefore, A20 does not only regulate NF-κB signaling in the context of normal immune responses, but it also appears to act as a tumor suppressor, regulating NF-κB signaling in the context of tumorigenesis.

## Mutations in Adaptors of TNF Signaling and Immune Dysfunction

Disease-associated mutations have also been identified in genes that encode target proteins of ubiquitination including *RIP1* and *NEMO*. Patients with autosomal recessive RIP1 deficiency are immunodeficient, as characterized by lymphopenia and recurrent infections, and develop inflammatory enterocolitis that resembles inflammatory bowel disease (IBD) (Cuchet-Lourenco et al., 2018; Abed et al., 2019; Uchiyama et al., 2019). Similar to patients with HOIP and HOIL-1L deficiencies, their fibroblasts had reduced MAPK and NF-κB signaling in response to TNF and polyinosinic:polycytidylic [poly(I:C)] (Cuchet-Lourenco et al., 2018). This was coupled with increased fibroblast death that appeared to be driven by necroptosis, as indicated by RIP3 and MLKL phosphorylation (Cuchet-Lourenco et al., 2018). However, while *ex vivo* stimulation of patients’ monocytes with LPS produced less IL-6, TNF, and IL-12 in response to LPS, they had increased IL-1β production (Cuchet-Lourenco et al., 2018), suggesting increased inflammasome activation. In addition to mutations that result in RIP1 deficiency, mutations in the caspase-8 cleavage site of RIP1 (D324) have also been identified in patients with periodic fevers and lymphadenopathy (Lalaoui et al., 2020; Tao et al., 2020). Peripheral blood mononuclear cells from these patients had enhanced susceptibility to both apoptotic and necroptotic stimuli, and increased pro-inflammatory cytokine production including IL-6, TNF, interferon-gamma, and IL-10 (Lalaoui et al., 2020; Tao et al., 2020). Together, these results highlight and validate RIP1’s unique physiological role in TNF signaling, as a mediator of pro-inflammatory signaling and as a regulator of cell death.

Mutations in *IKBKG*, the gene that encodes NEMO, are associated with both incontinentia pigmenti (Smahi et al., 2000) and X-linked recessive ectodermal dysplasia with immunodeficiency (Zonana et al., 2000; Doffinger et al., 2001). X-linked recessive ectodermal dysplasia with immunodeficiency is associated with hypomorphic mutations and the clinical phenotype is highly variable and may include recurrent infections, hyper-IgM levels, ectodermal dysplasia including coning teeth and hypodontia, inability to sweat, lymphedema, and osteopetrosis. This diverse presentation is due to both the variety of mutations that occur in these patients, but also the diversity of receptors associated with NF-κB signaling including ectodysplasin-A receptor, TNFR1, CD40, and receptor activator of NF-κB (RANK) (Zonana et al., 2000; Doffinger et al., 2001; Miot et al., 2017). While immunodeficiency due to both inadequate NF-κB mediated innate responses and CD40 signal in B cells is a primary medical concern in these patients, hematopoietic stem cell transplantation does not alleviate all of the associated disease. For instance, many patients have persistent colitis, even post-transplantation, which suggests epithelial specific defects are also important in the clinical phenotype (Miot et al., 2017).

## Deciphering TNF Signaling Regulation Through Genetic Mouse Models

Identification and characterization of patients with monogenic defects in TNF signaling components has provided critical insights into the significance of these proteins in regulating inflammatory signaling, and provides clinical context as to how dysfunction in this pathway can manifest in disease. While these clinical data are invaluable, there are experimental limits to what can be studied in patients and patient-derived samples. Therefore, spontaneous and genetically engineered mouse models have proven valuable tools to further interrogate TNF signaling pathways, to model diseases where these pathways likely play a role, and to identify how these pathways can be modulated when they go awry. A clear example of the value of these mouse models is the coincidental reporting of cleavage resistant *RIP1* mutations in patients with periodic fevers and the description of knock-in mice with complementary mutations (Newton et al., 2019; Zhang X. et al., 2019; Lalaoui et al., 2020; Tao et al., 2020). Genetic experiments in these mouse models demonstrated that observed clinical phenotypes were likely driven, at least in part, by TNFR1 and RIP1 kinase dependent apoptosis, but also highlight the complex role RIP1 plays in control both inflammatory and cell death pathways (Newton et al., 2019; Zhang X. et al., 2019; Lalaoui et al., 2020).

SHARPIN-deficient *cpdm* mice were the first LUBAC deficient mice characterized. SHARPIN deficiency results in reduced, but not eliminated, LUBAC activity and therefore is best characterized as a hypomorphic mouse (Seymour et al., 2007; Gerlach et al., 2011; Tokunaga et al., 2011). The most prominent phenotype in *cpdm* mice is eosinophilic dermatitis (HogenEsch et al., 1993) that begins around 1 week of age and progresses to severe disease by 6 weeks of age (Gijbels et al., 1996). Inflammatory infiltrates are also present in the joints, liver, and lung (Zhang et al., 2009) of these mice. Additionally, these mice develop eosinophilic esophagitis (Chien et al., 2015) and have hypoplastic lymphoid tissues (HogenEsch et al., 1999). While systemic immune infiltrates are partially dependent on lymphocytes, dermatitis in these mice is lymphocyte independent, indicating that this is an auto-inflammatory rather than autoimmune process (Potter et al., 2014). Loss of TNF or TNFR1 protects *cpdm* mice from both dermatitis and systemic inflammation, suggesting TNF signaling is the primary driver of inflammation (Gerlach et al., 2011; Kumari et al., 2014; Rickard et al., 2014). RIP1 kinase inhibition is also protective against dermatitis and reduces systemic inflammation in *cpdm* mice (Berger et al., 2014; Patel et al., 2020; Webster et al., 2020). *Cpdm* mice that express catalytically inactive RIP1^K45A^ do not develop dermatitis or systemic inflammation (Berger et al., 2014), and treatment with a RIP1 inhibitor, even starting at 6 weeks of age when there is disease induction, provides significant amelioration of the dermatitis and reduces immune infiltrates in the liver (Webster et al., 2020). Interestingly, while RIP3 loss delays the development of dermatitis in SHARPIN deficient mice (Kumari et al., 2014; Rickard et al., 2014), MLKL loss does not affect the development of dermatitis (Rickard et al., 2014). Consistent with this data, caspase-3 is robustly activated in the epidermis of SHARPIN deficient mice (Liang et al., 2011; Kumari et al., 2014; Rickard et al., 2014; Webster et al., 2020), while phosphorylated RIP3 positive cells were rarely detected in the dermis (Webster et al., 2020). Furthermore, the loss of 1 *caspase-8* allele in addition to RIP3 deficiency prevented the development of inflammatory lesions in most *cpdm* mice (Rickard et al., 2014). Together, this suggests that while RIP1 kinase activity drives the inflammation in *cpdm* mice, RIP1 is only partially signaling through RIP3 and the inflammation is primarily driven by apoptosis rather than necroptosis. Therefore, in some contexts, especially when epithelial barriers are disrupted, excessive apoptosis can be pro-inflammatory. Loss of caspase-1 also prevents the development of inflammatory lesions in c*pdm* mice. This protection is thought to be due to SHARPIN’s role in regulating caspase-1 activity in a LUBAC independent manner (Nastase et al., 2016).

Aside from the inflammatory lesions in the skin, joints and visceral organs, *cpdm* mice also have defective lymphoid development that includes altered splenic architecture and absence of Peyer’s patches (HogenEsch et al., 1999). Loss of TNF or TNFR1 does not restore the splenic architecture in *cpdm* mice (Gerlach et al., 2011; Kumari et al., 2014), but this is suspected to be partially due to the intrinsic defects in lymphoid development in the absence of TNFR1 signaling (Kumari et al., 2014). Similarly, caspase-1 deficient *cpdm* mice do not develop normal lymphoid architecture (Nastase et al., 2016). However, Peyer’s patches were restored in *Rip3^–/–^Casp8^±^ cpdm* mice (Rickard et al., 2014). The role of RIP1 kinase activity in the lymphoid phenotype of *cpdm* mice is not well characterized as evaluations of lymphoid tissues in *cpdm* with catalytically inactive RIP1^K45A^ have not been reported (Berger et al., 2014). While treatment with a RIP1 inhibitor after the onset of dermatitis did not restore the lymphoid architecture, this might be due to the late timing of the intervention and it is possible that germline loss of RIP1 kinase activity may restore the lymphoid architecture (Webster et al., 2020).

In contrast to the hypomorphic phenotype of *cpdm* mice, *Hoip* and *Hoil-1l* knock-out mice die around embryonic day 10.5 due to increased endothelial cell death and vascular collapse, most notably in the yolk sac (Peltzer et al., 2014; Peltzer et al., 2018). The timing of this is notable because this is also the stage when *Caspase-8* knock-out mice die due to RIP3 dependent necroptosis (Varfolomeev et al., 1998; Kaiser et al., 2011). While loss of caspase-8 and RIP3, loss of RIP1 catalytic activity due to the expression of catalytically inactive RIP1^K45A^, or loss of TNF signaling can extend survival to later embryonic stages in HOIL-1L deficient mice, only the combined loss of RIP1, RIP3, and caspase-8 is protective, which suggests cell death is a primary driver of embryonic lethality in these mice (Peltzer et al., 2018). Epidermal specific deletion of *Hoip* and *Hoil-1l* results in dermatitis in the perinatal period and death by post-partum day 6. Similar lesions are observed following inducible deletion of *Hoip* in adult mice (Taraborrelli et al., 2018). Dermatitis in epithelial-specific knock-out mice indicates that the inflammation is driven by an epithelial autonomous process, rather than being initiated by aberrant immune cell signaling. Similar to SHARPIN deficient mice, increased cell death, as evidenced by increased cleaved caspase-3 immunolabeling and terminal deoxynucleotidyl transferase dUTP nick end labeling (TUNEL), was observed in the epidermis of these mice. In germline epidermal specific *Hoip* and *Hoil-1l* knock-out mice, cell death is apparent at embryonic day 18.5 and precedes inflammatory cell infiltration into the dermis, suggesting that cell death is a cause rather than a consequence of inflammation. The significance of cell death in driving dermatitis is further supported by the fact that loss of caspase-8 and either MLKL or RIP3 is protective in these mice (Taraborrelli et al., 2018). Interestingly, while dermatitis appears to be solely driven by TNFR1 signaling in SHARPIN deficient mice, loss of TNFR1 only delays the onset of dermatitis to approximately 70 days, which appears to be due to concurrent signaling through other death receptors including TNF-related apoptosis inducing ligand (TRAIL) receptor and CD95. Additionally, in contrast to SHARPIN deficient mice, loss of RIP1 kinase activity through the expression of catalytically inactive RIP1^D138N^ does not show dramatic protection in these mice. However, the combination of small molecule RIP1 kinase inhibition and loss of TNFR1 expression provides more efficient protection in *Hoil-1l* knock-out mice, suggesting that RIP1 inhibition can provide a benefit independent of TNFR1 in some circumstances (Taraborrelli et al., 2018).

Both the similarities and differences between SHARPIN deficient mice that have hypomorphic LUBAC function and epidermal specific *Hoip* and *Hoil-1L* knock-out mice provide key insights into this pathway and its role in disease. Firstly, disruption in TNF stimulated linear ubiquitination can result in severe dermatitis. Cell death, predominantly apoptosis, is a key driver of inflammation in the skin of these mice, and inhibition of cell death can rescue the inflammation. This suggests that modulation of cell death pathways should be further considered for inflammatory skin diseases. Secondly, RIP1 inhibition was more effective when LUBAC activity was reduced rather than when it was eliminated (Berger et al., 2014; Taraborrelli et al., 2018). However, there was a benefit to RIP1 inhibition in addition to TNFR1 loss in the HOIP and HOIL-1L deficient mice (Taraborrelli et al., 2018), suggesting that the efficacy of RIP1 inhibition as a single agent might be context and disease specific. Additionally, the synergistic role of TNFR1 loss and RIP1 inhibition suggests that RIP1 inhibition is not just another means to disrupt the TNF signaling pathway, but scenarios where anti-TNFs and RIP1 inhibitors could be used in combination should be explored further.

Inducible inactivation of OTULIN’s DUB function in adult mice results in extensive hepatocyte and intestinal crypt cell death, and inflammation, primarily driven by myeloid cells, in the heart and liver (Heger et al., 2018). Similarly, co-deletion of *Birc2* and *Birc3*, which encode c-IAP1 and c-IAP2, respectively, in adult mice results in extensive hepatocyte death and crypt degeneration with intestinal villous atrophy and secondary inflammation (Zhang J. et al., 2019). In both OTULIN and c-IAP1/2 deficient mice, cell death in the liver and intestines was associated with extensive cleaved caspase-3 immunolabeling, suggesting a predominance of apoptosis (Heger et al., 2018; Zhang J. et al., 2019). While loss of RIP3 alone does not prevent lesions in either mouse, loss of caspase-8 and RIP3 rescued both the cell death and, to a significant degree, the associated inflammation. Since caspase-8 loss is embryonic lethal due to RIP3 mediated necroptosis, it is impossible to determine the independent contribution of apoptosis. However, the lack of protection with RIP3 loss alone and the extensive cleaved caspase-3 labeling suggests that apoptosis is the primary driver of the pro-inflammatory phenotype in these mice. In line with these observations in systemic, inducible *Otulin* or *Birc2/3* knock-out mice, hepatocyte specific deletion of *Otulin* results in hepatocyte apoptosis with resultant compensatory hyperplasia and inflammation that can progress to hepatocellular carcinoma (Damgaard et al., 2020; Verboom et al., 2020). Increased cell death and steatosis is evident in these mice by postnatal day 9. Interestingly, steatosis and increased liver enzymes were also identified in an OTULIN deficient patient (Damgaard et al., 2020). This hepatic injury can be alleviated by the loss of RIP1 kinase activity due to expression of the RIP1^D138N^ kinase dead protein, and more completely rescued by hepatocyte-specific *Fadd* deletion, indicating that the injury is driven by apoptosis signaling (Verboom et al., 2020), although loss of TNFR1 is not sufficient to protect against liver pathology in these mice (Damgaard et al., 2020). mTOR signaling is also increased in livers with hepatocyte-specific OTULIN deficiency. While treatment with the mTOR inhibitor rapamycin reduced the proliferative lesions and fibrosis in these mice, it did not reduce serum alanine aminotransferase (ALT) or aspartate aminotransferase (AST) levels, which suggests that while mTOR may be important for the proliferative response, it might not be the driver of the initial hepatocyte injury (Damgaard et al., 2020).

In contrast to germline loss of other components of the ubiquitin machinery, including LUBAC, OTULIN, and c-IAP1/2, that result in embryonic lethality, A20 deficient mice survive to birth, but subsequently develop multi-systemic inflammation that includes dermatitis, hepatitis, nephritis, enteritis, and arthritis (Lee et al., 2000). Inflammation in these mice appears to be lymphocyte independent because there was no protection when A20 deficient mice were crossed to *Rag1* knock-out mice (Lee et al., 2000). Consistent with their development of multi-systemic inflammation, *A20* knock-out mice have increased susceptibility to LPS and TNF, and this is associated with persistent NF-κB signaling in mouse embryonic fibroblasts (MEFs) (Lee et al., 2000). Loss of RIP3 or RIP1 kinase activity due the D138A kinase dead mutation significantly prolongs the survival of A20 deficient mice; however, a similar benefit is not observed in *Mlkl* knock-out mice (Onizawa et al., 2015; Newton et al., 2016). The difference in protection between RIP3 and MLKL deficient mice highlights the potential for necroptotic-independent functions of RIP3, which are not fully characterized.

## Dysregulation of TNF Signaling in Intestinal Inflammation and Arthritis

The role of TNF in inflammatory bowel disease and rheumatoid arthritis has been well established both in mouse models and in clinical practice (Williams et al., 1992; Elliott et al., 1993; Targan et al., 1997; Kontoyiannis et al., 1999). Constitutive TNF over-expression in the TNFΔARE mice, which have increased *Tnf* mRNA production and stability, develop Crohn’s-like ileitis that can progress to transmural and granulomatous inflammation and arthritis (Kontoyiannis et al., 1999). Ileitis even develops when TNF over-expression is restricted to intestinal enterocytes, although the disease onset and progression is delayed compared to mice with systemic TNF over-expression (Roulis et al., 2011; Bamias et al., 2013a). However, while TNF signaling in enterocytes causes apoptosis, it is not sufficient to cause ileitis, indicating the importance of paracrine signaling in other stromal and immune cells, rather than just enterocyte-restricted autocrine signaling (Roulis et al., 2011). Considering the permissive effects of TNF and the complexity of inflammatory bowel disease, it should not be surprising that disease progression requires an interplay of the epithelial, stromal, and hematopoietic compartments. Arthritis in TNFΔARE mice is characterized by synovial hyperplasia and myeloid infiltrates that progresses to cartilage and bone erosion and fibrosis, resulting in pannus (Kontoyiannis et al., 1999). Similarly, bone phenotype spontaneous mutation 1 (BPSM1) mice that have increased TNF expression due to a spontaneous insertion of a small interspersed element (SINE) in the 3’ untranslated region of *Tnf*, develop severe, progressive arthritis and valvular endocarditis with aortic aneurysm (Lacey et al., 2015). Development of arthritis requires local TNF production, as evidenced by the lack of joint changes in TNFΔARE mice with intestinal-specific TNF hyper-secretion (Bamias et al., 2013b). In BPSM1 mice, bone marrow transplantation of wild-type or BPSM1 cells and genetic crosses with *Tnfr1^–/–^* mice suggest that while myeloid cells are necessary for TNF production in this model, TNFR1 signaling on non-hematopoietic cells, presumable synoviocytes, is required for the development of arthritis (Lacey et al., 2015). TNF blockade is also protective in collagen-induced and anti-collagen antibody-induced arthritis models (Williams et al., 1992; Patel et al., 2020), and this is consistent with the clinical benefit of TNF blockade in rheumatoid arthritis patients. While RIP1 inhibition provided a similar benefit in anti-collagen antibody-induced arthritis model, there was no synergistic benefit in combining TNF and RIP1 inhibition (Patel et al., 2020). This is in contrast to the added protective benefit of combinatorial blockade in the development of dermatitis in LUBAC deficient mice (Taraborrelli et al., 2018). Therefore, the benefit of combination therapies is likely to be context specific and requires further exploration.

Patients with hypomorphic *NEMO* mutations frequently develop colitis. The fact that this colitis is not responsive to hematopoietic stem cell transplantation suggests that NEMO deficiency has cell autonomous effects in intestinal enterocytes (Miot et al., 2017). This has been studied in mice by using a Cre recombinase driven by the *villin* promoter to specifically delete *Nemo* from intestinal epithelial cells. Enterocyte-specific NEMO loss results in severe colitis, particularly in the proximal colon, and small intestinal crypt cell death with Paneth cell loss (Nenci et al., 2007; Vlantis et al., 2016). While TNFR1 loss and germ-free conditions protect against colitis, increased cell death in the small intestine remains (Vlantis et al., 2016). Similar to *SHARPIN*-mutant mice, RIP3 loss affords inconsistent and incomplete protection in NEMO deficient mice, while inactivation of RIP1 kinase activity via RIP1^D138N^ or RIP3 and FADD combined ablation provide complete protection (Vlantis et al., 2016). Pharmacologic RIP1 inhibition is similarly fully protective in these mice (Patel et al., 2020). Again, this suggests that RIP1 mediated apoptosis can drive both extensive tissue damage and inflammation in the context of dysfunctional TNF signaling.

While TNF signaling is biased toward cell death pathways in NEMO deficient mice, presumably in part due to a lack of NF-κB signaling, enterocytes with overactive NF-κB signaling are also sensitive to TNF-induced cell death. IKKβ (EE)^IEC^ mice have constitutive NF-κB signaling in intestinal epithelial cells (Guma et al., 2011). These mice have increased sensitivity to LPS due to MAPK mediated TNF production (Guma et al., 2011). TNF stimulation in enteroids from these mice causes intestinal epithelial cell apoptosis, as noted by increased cleaved caspase-3 and caspase-8. Genetic loss of RIP1 catalytic activity through expression of RIP1^D138N^ protected enterocytes from TNF induced apoptosis, while RIP3 loss was not protective. Similarly, both genetic and pharmacologic RIP1 inactivation protected these mice from LPS induced intestinal cell death *in vivo* (Wong et al., 2020). Together, the increased susceptibility of both the IKKβ (EE)^IEC^ mice and NEMO deficient mice to TNF-induced apoptosis suggests that NF-κB signaling needs to be tightly controlled and dysregulation in either direction may shift TNF signaling from a pro-survival to a pro-death pathway. Interestingly, RIP1 kinase activity is a potent driver of cell death in both scenarios. This further strengthens the hypothesis that RIP1 inhibition may provide a therapeutic benefit to IBD patients.

The *ATG16L1^*T*300A^* polymorphism is associated with Crohn’s disease, and ATG16L1 has an important role in Paneth cell survival and function (Cadwell et al., 2008). Norovirus infected mice with reduced ATG16L1 expression have decreased and disorganized Paneth cell granules and decreased lysozyme, and similar defects have been identified in Crohn’s disease patients (Cadwell et al., 2008, 2010). ATG16L1 deficient mice also have increased susceptibility to dextran sodium sulfate (DSS)-induced colitis and, in the presence of norovirus infection, develop small intestinal villous atrophy and have loss of Paneth cells. This small intestinal pathology is driven by increased epithelial TNF production and subsequent cell death, and is protected by RIP1 kinase inhibition (Matsuzawa-Ishimoto et al., 2017). Increased Paneth cell death has been identified in the ileum of Crohn’s disease patients, and treatment of control patient biopsies with TNF has been shown to reduce Paneth cell-associated *lysozyme* mRNA, which can be rescued by Nec-1, a RIP1 inhibitor (Gunther et al., 2011). Considering the importance of Paneth cells in producing anti-microbial peptides and innate immune responses in the intestine, TNF mediated Paneth cell death may play an important role in the pathogenesis of Crohn’s disease. Given the protection observed with RIP1 inhibitors in the survival of mouse and human Paneth cells, and the intrinsic role of RIP1 kinase activity in intestinal pathology secondary to NFκB dysregulation, RIP1 inhibitors should be further evaluated in the treatment of inflammatory bowel disease.

## RIP1 Inhibitors for Treatment of TNF Mediated Inflammatory Diseases

While TNF inhibition is efficacious in the treatment of many inflammatory disease, it is also associated with immunosuppression and increased risk of infections, and many patients are refractory to TNF inhibitors (Taylor and Feldmann, 2009; Adegbola et al., 2018). RIP1 inhibition may provide an alternative mechanism to treat inflammatory diseases with no known risk of immunosuppression (Shan et al., 2018; Yuan et al., 2019). While the *Rip1* knock-out mouse dies in the perinatal period due to RIP3 mediated inflammation and caspase 8 mediated intestinal apoptosis (Kelliher et al., 1998; Dillon et al., 2014; Kaiser et al., 2014), catalytically dead *Rip1* knock-in (RIP1 KD) mice are viable and healthy, even when aged to 18 months (Berger et al., 2014; Kaiser et al., 2014; Newton et al., 2014; Polykratis et al., 2014; Webster et al., 2020). RIP1^D138N^ KD mice were able to clear both vaccinia virus and mouse gammaherpesvirus, MHV68, at a similar rate compared to wild-type mice and these mice showed no immunologic dysfunction following MHV68 infection (Webster et al., 2020). This suggests that while RIP1 scaffolding functions are essential for survival, RIP1 kinase activity can be inhibited without detrimental effects.

As described above, RIP1 kinase inhibition is protective against inflammation in the skin, intestines, and joints secondary to dysfunctions in TNF and NF-κB signaling (Berger et al., 2014; Vlantis et al., 2016; Patel et al., 2020; Webster et al., 2020; Wong et al., 2020). Patients with mutations in these pathways have variably responded to different biologics including anti-IL-1, anti-TNF, and anti-IL-6 molecules (Damgaard et al., 2016; Zhou et al., 2016b; Lalaoui et al., 2020; Tao et al., 2020). It still is to be seen whether these patients would benefit from RIP1 inhibitors. The role of RIP1 kinase activity in inflammation is also evident in the TNF-induced systemic inflammatory response syndrome (SIRS) model, in which genetic or pharmacologic RIP1 inhibition is protective (Newton et al., 2014; Polykratis et al., 2014; Newton et al., 2016; Patel et al., 2020). Interestingly, in some disease models, such as anti-collagen antibody-induced arthritis, combined RIP1 and TNF inhibition does not show a synergistic effect suggesting these proteins are working on a linear pathway (Patel et al., 2020). However, in other models, such as HOIP and HOIL-1L deficient mice, TNF and RIP1 inhibition plays a synergistic role (Taraborrelli et al., 2018), indicating that combination therapies might be efficacious in some diseases. Although RIP1 has been implicated in numerous disease models, the results have not always been reproducible (Newton et al., 2016; Patel et al., 2020; Webster et al., 2020). Therefore, more studies are needed to define the context and potential combination therapies that will provide the maximal benefit for RIP1 inhibition. However, the true test of RIP1 inhibition in inflammatory diseases will be in clinical trials.

To date, GlaxoSmithKline (GSK) and Denali have tested their RIP1 inhibitors in clinical settings and reported that GSK2982772 and DNL104 were generally well tolerated in human subjects (Harris et al., 2017; Weisel et al., 2017; Grievink et al., 2020; Jensen et al., 2020; Martens S. et al., 2020). Denali’s brain-penetrant RIP1 inhibitor DNL104 did not cause any central nervous system toxicities but 37% percent of subjects receiving multiple doses of DNL104 had post-dose liver toxicity (Grievink et al., 2020). Denali has, in the meantime, terminated clinical examination of DNL104 and in collaboration with Sanofi entered another RIP1 inhibitor, DNL747, in clinical trials for Alzheimer’s disease, amylotrophic lateral sclerosis, and multiple sclerosis (Jensen et al., 2020; Martens S. et al., 2020). GSK2982772 is a systemic, non-brain penetrant RIP1 inhibitor was well-tolerated with no serious adverse events (AEs) and no suggestion of a safety concern (Weisel et al., 2017). Encouraged by favorable safety data, GSK has entered GSK2982772 into small phase 2 clinical trials for psoriasis, rheumatoid arthritis, and ulcerative colitis. So far, GSK2982772 has not shown significant therapeutic benefit in psoriasis or rheumatoid arthritis (clinicatrials.gov), while the data from the ulcerative colitis trial are still pending. GSK has also ventured into cancer trials with a different RIP1 inhibitor, GSK3145095 (Harris et al., 2019). However, that particular trial, designed to test the ability of RIP1 inhibitor to provide benefit in pancreatic and other solid tumors, was relatively quickly terminated (Martens S. et al., 2020). This may not come as a complete surprise given that protective role of RIP1 inhibition in pancreatic cancer was never fully validated (Patel et al., 2020). Thus, although RIP1 inhibition presents an attractive opportunity to target TNF mediated inflammatory diseases, further efforts are needed to fully explore this therapeutic strategy.

## Author Contributions

JW and DV wrote and contributed to this manuscript and approved this submission.

## Conflict of Interest

JW and DV were employees and shareholders at Genentech-Roche.
